# DNA barcoding uncovers cryptic diversity in 50% of deep-sea Antarctic polychaetes

**DOI:** 10.1098/rsos.160432

**Published:** 2016-11-02

**Authors:** Madeleine J. Brasier, Helena Wiklund, Lenka Neal, Rachel Jeffreys, Katrin Linse, Henry Ruhl, Adrian G. Glover

**Affiliations:** 1 School of Environmental Science, University of Liverpool, L69 3BX, Liverpool, UK; 2 Life Sciences, Natural History Museum, Cromwell Road, London SW7 5BD, UK; 3 BioSciences, British Antarctic Survey, Cambridge CB3 OET, UK; 4 National Oceanography Centre, University of Southampton, Waterfront Campus, Southampton SO14 3ZH, UK

**Keywords:** Southern Ocean, benthos, COI, 16S, species diversity

## Abstract

The Antarctic marine environment is a diverse ecosystem currently experiencing some of the fastest rates of climatic change. The documentation and management of these changes requires accurate estimates of species diversity. Recently, there has been an increased recognition of the abundance and importance of cryptic species, i.e. those that are morphologically identical but genetically distinct. This article presents the largest genetic investigation into the prevalence of cryptic polychaete species within the deep Antarctic benthos to date. We uncover cryptic diversity in 50% of the 15 morphospecies targeted through the comparison of mitochondrial DNA sequences, as well as 10 previously overlooked morphospecies, increasing the total species richness in the sample by 233%. Our ability to describe universal rules for the detection of cryptic species within polychaetes, or normalization to expected number of species based on genetic data is prevented by taxon-specific differences in phylogenetic outputs and genetic variation between and within potential cryptic species. These data provide the foundation for biogeographic and functional analysis that will provide insight into the drivers of species diversity and its role in ecosystem function.

## 1. Introduction

Antarctica is a fragile environment currently undergoing some of the fastest rates of climatic change on the planet [1,2]. These changes are predicted to have a significant impact on its marine communities if species are unable to adapt to their new conditions [3,4]. In order to detect, document and manage the impact of environmental change our knowledge of species diversity needs to be improved. For these reasons there has been an increased effort to accurately document and assess current species diversity within Antarctic waters. A major contributor in this were the Census of Antarctic Marine Life (CAML; http://www.caml.aq) and the Census of Diversity of Abyssal Marine Life (CeDAMar: http://www.coml.org/projects/census-diversity-abyssal-marine-life-cedamar) campaigns [5]. This project led to substantial systematic investigations into the biodiversity and biogeography of marine animals within the Southern Ocean and Antarctic Islands using both traditional and molecular methods of species identification. Until relatively recently, there were limited DNA barcodes, short sequences of DNA from a single organism, available for Antarctic marine species. In 2009, Grant & Linse [6] documented that genetic data were only available for 2.6% of marine invertebrate species. Although the number of Antarctic DNA barcodes is rising, increasing from 432 to 20 355 between 2009 and 2011 [7], the majority of these sequences originate from Molluscan and Crustacean species collected from the Weddell Sea and the Antarctic Peninsula.

Accurate documentation of species diversity is the primary step in understanding the patterns and controls of diversity levels, biogeography and functional ecology—all of which are fundamentally important to the management of marine ecosystems. Ecosystem-based management has been practised in Antarctica to regulate its fisheries since the early 1980s regulated by the Commission for the Conservation of Antarctic Marine Living Resources (CCAMLR) [8]. For management practices to be most effective, they should be based on sound scientific research and high-quality data. For example, the assignment of any marine protected areas should be based on a combination of diversity, productivity, bathymetric and habitat data to ensure protection of the most ecologically important or vulnerable locations in a variety of settings. The capacity of managing human impacts on Antarctic marine ecosystems can thus increase by continuing the research objectives set out by CAML [9,10].

The use of DNA barcodes to identify Antarctic fauna has uncovered previously overlooked cryptic species, those that are morphologically indistinguishable but genetically distinct, which appear to be a common feature within the Antarctic benthos [7]. Genetic analysis has also identified areas within the Southern Ocean such as the Scotia Arc as potential hotspots of cryptic diversity [11]. This is perhaps not surprising given the isolated nature and glacial history of Antarctic waters, which could have separated populations, promoting genetic divergence and cryptic speciation by reproductive isolation [11]. It has been proposed that the ecological impacts of repeated glacial and interglacial cycles could act as a speciation driver [12,13]. Ice advances during glacial maxima physically remove most of the marine benthos inhabiting the continental shelf by depositing it on to the continental slope within glacial debris. Thus, for species to persist through these glacial periods they would have had to survive within the deep sea or have shelf refugia within areas of no sea ice, such as polynyas [14,15]. During glacial maxima gene flow between populations would have been lower leading to increased genetic variation between populations. Under extreme environmental conditions, there may be increased selection pressures on behaviour and physiological character rather than functionality, thus reducing or eliminating morphological changes that can accompany speciation [16]. So assuming the functionality of the isolated populations remained constant, it is probably that their morphology would have gone unchanged, potentially resulting in high levels of cryptic species. To date, evidence of cryptic species has been documented in several Antarctic marine taxa including crustaceans [17,18], molluscs [11,19], polychaetes [20,21], echinoderms [22,23] and nemerteans [24].

Polychaetes represent one of the dominant taxa in benthic marine communities including Antarctic waters where they can account for more than 70% of macrofauna (organisms retained in a 300 µm sieve) [25,26] with recorded abundances of more than 300 individuals per 0.1 m^2^ [27]. As discussed in a recent review by Nygren [28], there is evidence to suggest that cryptic species are common among all polychaete families, making up a significant portion of their biodiversity. Whether or not cryptic species are more prevalent within certain polychaete families, functional groups or environments is, however, unknown. For this reason, the use of molecular methods for accurate identification of morphologically distinct and cryptic polychaete species is essential if we are to understand their true diversity. The first major comprehensive DNA barcoding project of polychaetes was conducted by Carr *et al*. [29], who sequenced 1876 specimens from waters surrounding Alaska and the Canadian Artic. In total, 25% of the morphospecies examined were composed of two or more distinct genetic lineages and deemed to contain cryptic species. Results such as this suggest that polychaete identification based on morphological characters alone may significantly underestimate species diversity.

Here, we present mitochrondrial DNA sequences from 15 polychaete morphospecies collected during British Antarctic Survey (BAS) expeditions in the western Antarctic region including the Scotia Arc, the Amundsen Sea Embayment and Pine Island Bay and the southeastern Weddell Sea. Using DNA sequences of two mitochondrial DNA loci, we assess the level of cryptic diversity of polychaetes. More specifically, we use these data to (i) re-evaluate levels of species diversity in Southern Ocean polychaetes using molecular techniques, (ii) discuss whether general rules can be applied to detect cryptic species in polychaetes, e.g. is there a consistent level of genetic difference between cryptic species that could be used to identify them in the future, and (iii) compare the use of the mitochondrial COI and 16S regions as barcoding genes for polychaetes. We use these findings to evaluate our overarching hypothesis that the level of polychaete diversity within the Southern Ocean is currently underestimated based on morphological species identification.

## 2. Material and methods

### 2.1. Sample collection

**Figure 1.**
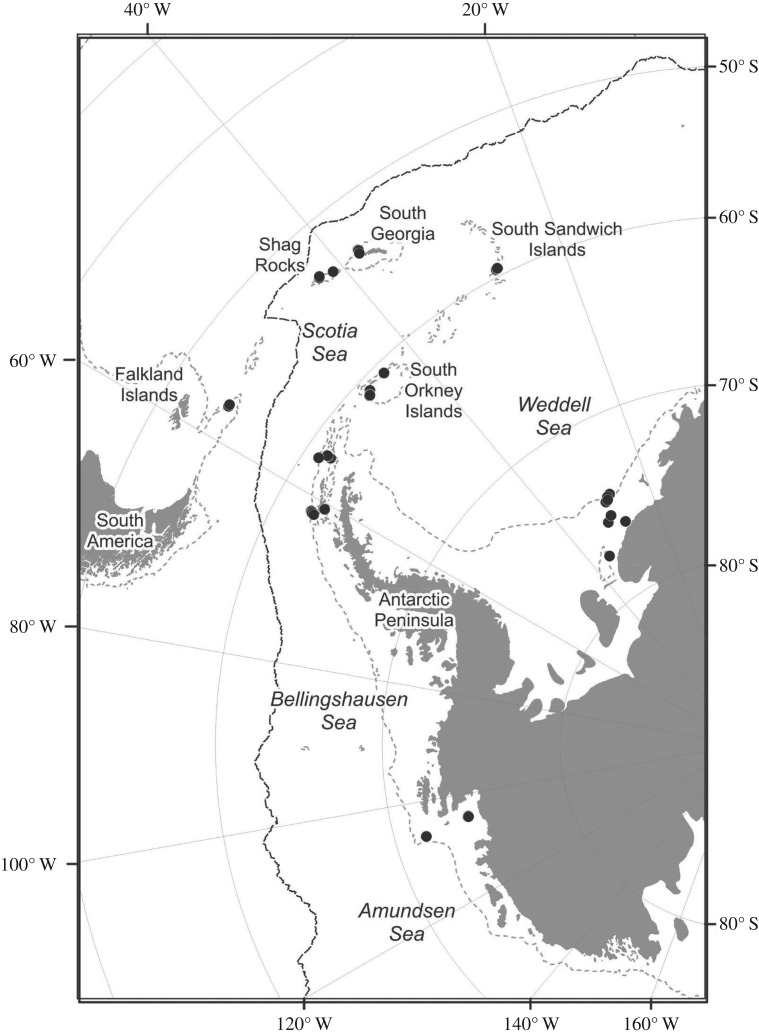
Location of the epibenthic sledge and Agassiz trawl stations of the BIOPEARL I (Scotia Sea), BIOPEARL II (Amundsen Sea) and JR275 (Weddell Sea) cruises from which target species were collected and barcoded.

Specimens were collected using both an epibenthic sledge (EBS) and Agassiz trawl (AGT), between depths of 100 to 3500 m during expeditions JR144, JR179 (BIOPEARL I and II, Biodiversity Dynamics: Phylogeography, Evolution and Radiation of Life) and JR275 with RRS *James Clark Ross* (JR). Specific sampling protocols and preservation procedures used are described in Neal *et al*. [20] for EBS and Griffiths *et al*. [30] for AGT. Specimens from a total of 16 sites across the western Antarctic area were used including 6 sites within the Scotia Arc (BIOPEARL I), 4 sites within the Amundsen Sea (BIOPEARL II) and 6 sites within the Weddell Sea (JR275), (figure 1).

### 2.2. Morphological species identification

All individual polychaetes collected during BIOPEARL I and II and individuals of the selected target species from JR275 were identified from morphological characters by the same taxonomist. Where possible, individuals were assigned to named species using published dichotomous keys; however, in many cases species lacked description and were assigned a morphological operational taxonomic unit at the highest identifiable taxonomic level, i.e. some could be resolved to genus level, e.g. *Flabelligena* sp. A and *Flabelligena* sp. B, whereas others were only identifiable to family level, e.g. Polynoidae sp. A.

### 2.3. Specimen selection for DNA barcoding

**Figure 2.**
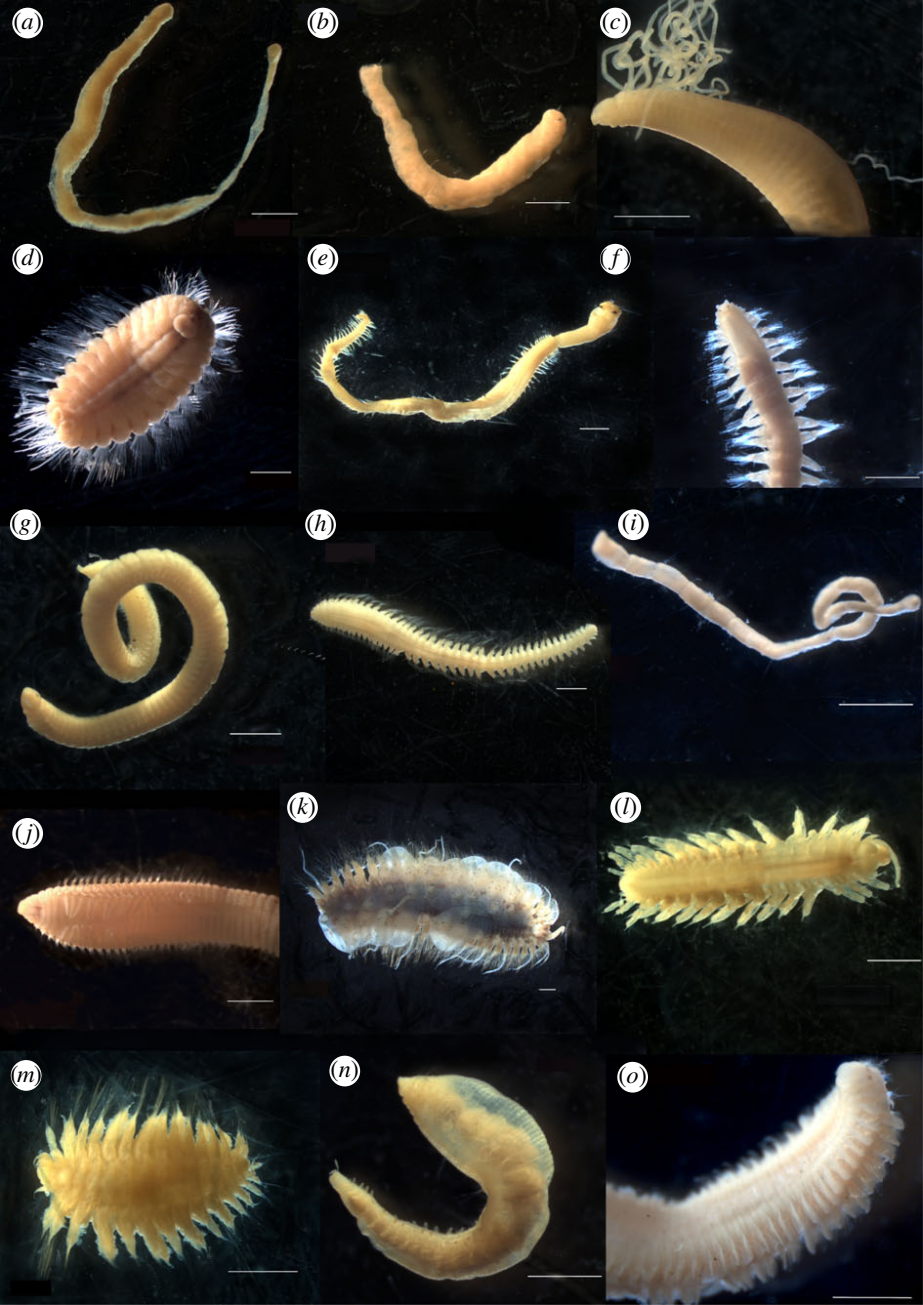
Photos of the 15 original target morphospecies selected for DNA barcoding, scale bars, 1000 µm. In alphabetical order by family (*a*) *Flabelligena* sp. A, (*b*) *Flabelligena* sp. B (Acrocirridae), (*c*) *Chaetozone* sp. A (Cirratulidae), (*d*) *Euphrosinella cirratoformis* (Euphrosinidae), (*e*) *Glycera capitata* (Glyceridae), (*f*) Hesionidae sp. A (Hesionidae), (*g*) *Lumbrineris kerguelensis-cingulata* (Lumbrineridae)*,* (*h*) *Maldane sarsi* (Maldanidae), (*i*) *Aglaophamus trissophyllus* (Nephtyidae), (*j*) *Aricidea simplex* (Paraonidae), (*k*) *Harmothoe fuligineum*, (*l*) *Macellicephala* sp. A, (*m*) *Macellicephaloides* sp. B (Polynoidae), (*n*) *Scalibregma inflatum* (Scalibregmatidae) and (*o*) *Laonice weddellia* (Spionidae).

The selection of target species for DNA barcoding was a non-random process; it was based on an informed combination of methodological requirements and research considerations. Approximately half the BIOPEARL polychaete individuals were fixed in formalin, which is known to denature DNA. Thus, those preserved in ethanol were chosen in order to limit preservation effects of sequence quality. The next major consideration when choosing target species was numbers of specimens. As multiple individuals are needed to detect cryptic species, we excluded all species with less than 10 individuals preserved in ethanol. From the remaining individuals, target species were chosen based on their taxonomic groups, functional traits and biogeographic distributions. We aimed to cover a range of these criteria as current knowledge on the prevalence of cryptic species across polychaete families and functional groups is limited. In total, 15 polychaete morphospecies (figure 2) were selected from the 400 available covering 12 out of the 28 families present in the sample set.

### 2.4. DNA extraction and sequencing

The selection of the part of each specimen to dissect for DNA extraction varied between families depending on their most useful taxonomic characteristics, in order to allow for possible re-examination of specimen morphology after DNA sequencing. For example, parapodia were taken from Polynoidae specimens, mid-body segments from Glyceridae and ventral tissue from Nephtyidae. DNA was extracted using a Qiagen DNeasy Blood and Tissue kit. Of the 463 DNA extractions, 131 were extracted using individual spin columns following the protocol provided by the manufacturer and the remaining 332 extracted by the Natural History Museum Sequencing Facility using a Hamilton Microlab STAR Robotic Workstation.

Part of the mitochondrial protein-coding COI (the so-called ‘Folmer fragment’, around 660 bp) gene was the primary gene targeted for this project. The COI gene was chosen as it is the gene required for formal barcode status on the Barcode of Life Data System (BOLD). The COI gene is a suitable barcoding gene as it is fast evolving and exhibits a greater degree of genetic distance between than within species [31]. However, with the increase in sequencing projects across all taxa, it is now becoming apparent that COI is not always the most attainable, and other mitochondrial genes can be used. Following variable PCR success with COI primers in this project for many target species the non-coding mitochondrial 16S rDNA gene (around 500 bp) was also targeted. This gene can be used in a similar way to COI for species discrimination [32,33], it is often easier to obtain and, in the case of Antarctic invertebrates, most widely available [6].

DNA extractions were amplified using a PCR mix of 21 µl Red Taq DNA Polymerase 1.1X MasterMix (VWR), 1 µl of each primer (10 µM) and 2–5 µl of DNA extract. The PCR temperature profile consisted of an initial 5 min denaturation stage at 95°C, followed by 35 cycles of 95°C denaturation for 1 min, 55°C annealing for 1 min, 74°C extension for 2 min with an additional 5 min extension phase after the last cycle. For primer sequences and references, see table 1. PCR products were purified using a Millipore Multiscreen 96-well PCR purification system and sequenced on an ABI 3730XL DNA Analyser (Applied Biosystems) at the Natural History Museum Sequencing Facility using the same primers as in the PCR.

**Table 1.** COI and 16S primers used for PCR of the polychaete DNA.

primer name	sequence (5′-3′)	reference
LCO	GGTCAACAAATCATAAAGATATTGG	Folmer *et al*. [34]
HCO	TAAACTTCAGGGTGACCAAAAA ATCA	Folmer *et al*. [34]
CO1-E	TATACTTCTGGGTGTCCGAAGAATCA	Carr *et al*. [29]
polyLCO (F)	GAYTATWTTCAACAAATCATAAAGATATTGG	Carr *et al*. [29]
polyHCO (R)	TAMACTTCWGGGTGACCAAARAATCA	Carr *et al*. [29]
polyshortCOIR (R)	CCNCCTCCNGCWGGRTCRAARAA	Carr *et al*. [29]
Ann16Sr	TCCTAAGCCAACATCGAGGTGCCAA	Sjölin *et al*. [36]
Ann16Sf	GCGGTATCCTGACCGTRCWAAGGTA	Sjölin *et al*. [36]

### 2.5. Sequence analysis

Overlapping sequences (from forward and reverse primers) were assembled into consensus sequences and aligned in Geneious. 7.1.4 [37]. For phylogenetic analysis, additional sequences from the same, or when limited, closely related families, were downloaded from GenBank (http://www.ncbi.nlm.nih.gov/genbank/). For some species, additional COI sequences were also included from private databases within BOLD (http://www.barcodeoflife.org/). COI sequences were aligned using MUSCLE [38] and 16S using MAFTT [39] both using the default settings and provided as plug-ins in Geneious. At least one outgroup was chosen for each alignment; the species were selected from either a sister taxa or family within the same order. If available the choice of outgroups for some families was also inferred from previously published phylogenies.

Bayesian phylogenetic analyses were conducted for each morphospecies investigated using the separate 16S dataset and, where possible, the separate COI dataset. For each dataset, the best nucleotide substitution model was chosen using the jModelTest Akaike and Bayesian information criterion [40]. Either GTR + I + G or GTR + G models were chosen as the best-fit model for each alignment. All analyses were run three times for 10 000 000 generations using MrBayes v. 3.1.2 [41] with 2 500 000 generations discarded as burn-in. All phylogenetic trees were edited in FigTree v. 1.4 [42] and Adobe Illustrator CS5.1.

The inclusion of publicly available sequences of closely related species allowed the comparison of genetic distances between potential cryptic species and known morphologically described species. Thus, if the genetic distances were greater than or comparable to the genetic distances between known species, this provided evidence for the presence of cryptic species. For this, the Kimura's two-parameter substitution model (K2P) [43] was calculated using Mesquite [44] for pairwise comparisons of sequence divergence within and between species based on the number of nucleotide substitutions.

### 2.6. Secondary morphological analysis

Following DNA analysis, all individuals within the same morphospecies that appeared to be genetically distinct from one another were re-examined. Some specimens were also sent to taxonomic specialists, for example the targeted morphospecies within the families Nephtyidae and Maldanidae. Following both sequence and secondary morphological analysis, some specimens were reassigned to different morphospecies including both described species and undescribed morphospecies, while others were considered to still be true cryptic species in which clear morphological differences were not easily identified.

### 2.7. Operational taxonomic units

Throughout our analyses a phylogenetic species concept was used. This works on the principle that the genetic variation between species (interspecific) is greater than the genetic variation within species (intraspecific) [45]. Thus, where two or more species are distinct, there should be a lack of overlap between intraspecific and interspecific sequence variation, commonly referred to as the ‘barcoding gap’ [46]. Potential cryptic species were identified based on phylogenetic analysis; cryptic clades were determined from tree topography and the clade formation of the sequenced morphospecies in comparison to other known morphospecies and cryptic species within each family. K2P percentages were used as a secondary tool for identification, comparing interspecific and intraspecific genetic variation and the existence of a ‘barcoding gap’ once clades were determined.

All potential species (both morphological and cryptic) were labelled with the first author's initials (MB), if multiple species were found they were also assigned a unique number. Species that exhibited multiple clades which could not be resolved owing to high intraspecific variation, were considered to be a ‘species complex’ as well-supported phylogenetic species could not be resolved. Clades within a species complex were assigned the same MB# with an additional letter specific to their clade. For example, MB1a and MB1b would be different clades of the same species, while MB1 and MB2 are two separate cryptic species.

## 3. Results

**Figure 3.**
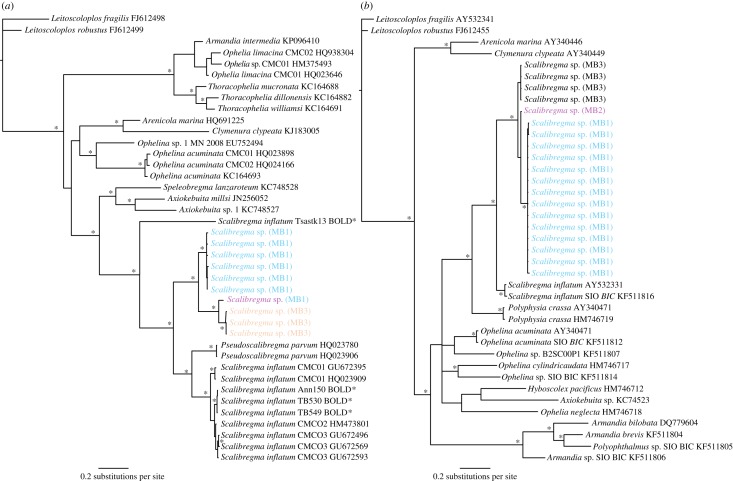
Phylogenetic tree of Scalibregmatidae from Bayesian analysis using COI (*a*) and 16S (*b*). An example of results ‘scenario 1’, evidence for cryptic diversity in COI and 16S genes, cryptic species *Scalibregma* sp. (MB1), (MB2) and (MB3). Outgroup: *Leitoscoloplus fragilis* and *L. robustus* (Orbiniidae), asterisk indicates significant node values (more than 95%) for Bayesian posterior probabilities. BOLD* indicates sequences obtained from a private BOLD database.

**Figure 4.**
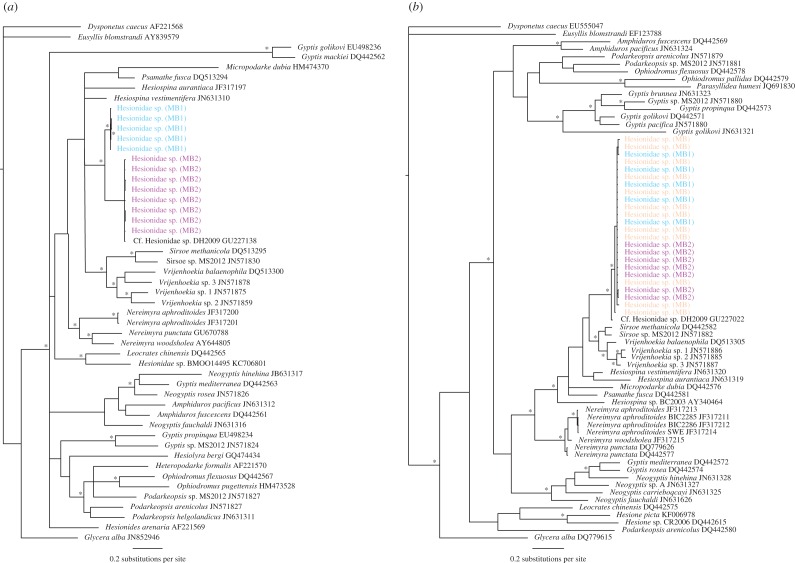
Phylogenetic tree of Hesionidae from Bayesian analysis using COI (*a*), the corresponding 16S sequences (*b*). An example of results ‘scenario 2’, evidence for cryptic diversity in COI but not 16S. Cryptic species are labelled Hesionidae sp. (MB1) and Hesionidae sp. (MB2) and the unassigned specimens based on 16S data only labelled Hesionidae sp. (MB). Outgroups: *Dysponetus caecus* (Chrysopetalidae) and *Eusyllis blomstrandi* (Syllidae), asterisk indicates significant node values (more than 95%) for Bayesian posterior probabilities.

**Figure 5.**
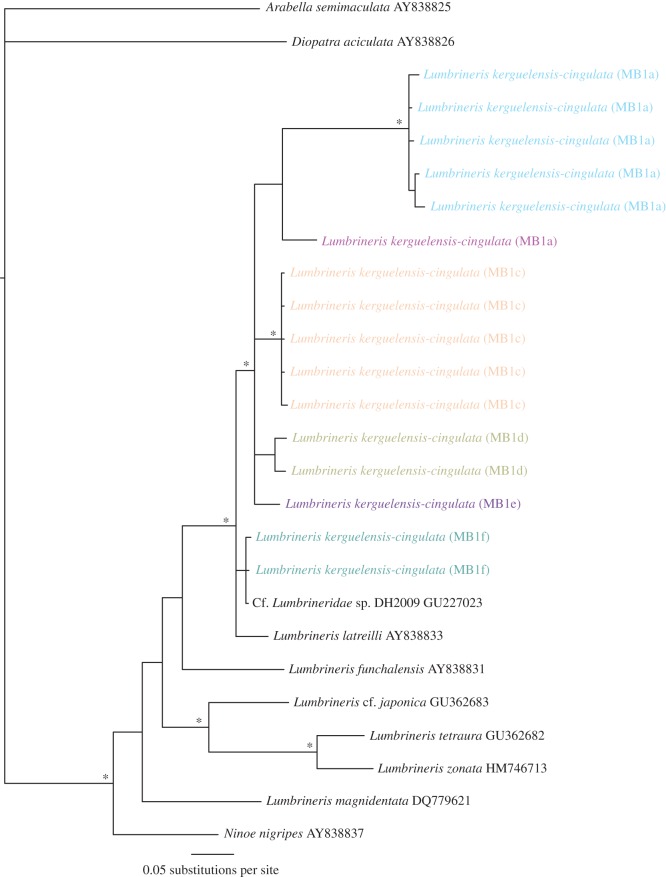
Phylogenetic tree of Lumbrineridae from Bayesian analysis using 16S only. An example of results ‘scenario 3’, an unresolved species complex as a result of high intraspecific variation and morphological uncertainty. Clades are labelled with unique MB1 letters. Outgroups: *Arabella semimaculata* (Oenonidae) and *Diopatra aciculata* (Onuphidae), asterisk indicates significant node values (more than 95%) for Bayesian posterior probabilities.

In general, the phylogenetic results followed one of four scenarios: (1) evidence of cryptic species based on coherent COI and 16S phylogenies or if COI was not sequenced just 16S data, e.g. *Scalibregma inflatum* (figure 3); (2) evidence of cryptic species in the COI phylogeny but not 16S, e.g. Hesionidae sp. A (figure 4); (3) undetermined clades from 16S analysis, in this case we were unable to distinguish between the presence of potential cryptic species or high intraspecific variation within a species complex, e.g. *Lumbrineris kerguelensis-cingulata* (figure 5); (4) no evidence of cryptic species, e.g. *Laonice weddellia*.

### 3.1. Evidence of cryptic species based on coherent COI and 16S phylogenies

**Figure 6.**
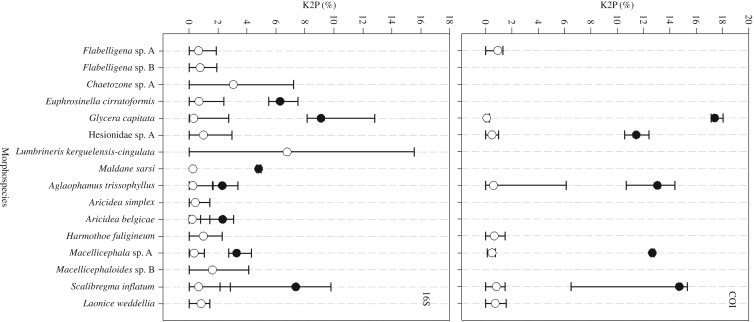
Average pairwise K2P distance (%) as a measure of interspecific variation between the cryptic clades (black circles) and intraspecific variation within clades (white circles) identified from phylogenetic analysis. Note that COI data were not available for all species. Additionally, for Hesionidae sp. A, the cryptic clades identified from the COI phylogeny could not be determined from 16S (figure 4), thus there is no measure of interspecific variation for this gene.

The presence of 10 potential cryptic species was recorded within eight of the 15 morphospecies sequenced. In six cases, these could be identified using both COI and 16S phylogenies. These included *Glycera capitata, Scalibregma inflatum, Macellicephala* sp. A, *Aricidea belgicae* (previously identified as *A. simplex*), *Euphrosinella cirratoformis* and *Maldane sarsi*. The number of cryptic species uncovered within each morphospecies ranged from one to three (table 2), and the majority of the cryptic species in this study were co-occurring within the same localities. For *G. capitata, S. inflatum* (figure 3) and *Macellicephala* sp. A, evidence of cryptic diversity could be identified from both COI and 16S phylogenetic and distance analysis. In each of these cases, the clade groupings were consistent across the two genes and the intraspecific and interspecific variation inferred from the K2P distance percentage was consistently greater in COI than 16S (figure 6).

**Table 2.** Primary identification of each morphospecies using light microscopy with their secondary identification based on phylogenetic analysis and morphological re-examination. This confirmed the presence or absence of cryptic species, which is also listed. All species were assigned a unique MB# and species complexes containing multiple clades were assigned the same MB# with an additional letter. The number of COI and 16S sequences obtained for each species, merged 16S cells, indicates inability to distinguish between species using this gene.

primary species identification	secondary species identification	evidence of cryptic species	number of COI sequences	number of 16S sequences	GenBank accession numbers (COI)	GenBank accession numbers (16S)
*Flabelligena* sp. A (Acrocirridae)	*Flabelligena* sp. A (MB)	no	8	12	KX867405-7412	KX867212-7223
*Flabelligena* sp. B (Acrocirridae)	*Flabelligena* sp. B (MB)	no	2	8	KX867413-7414	KX867224-7231
*Chaetozone* sp*.* A (Cirratulidae)	*Chaetozone* sp. (MB1a)	no	—	7	—	KX867175-7181
	*Chaetozone* sp. (MB1b)		—	1	—	KX867182
	*Chaetozone* sp. (MB1c)		—	2	—	KX867183-7184
*Euphrosinella cirratoformis* (Euphrosinidae)	*Euphrosinella* cf. *cirratoformis* (MB1)	yes	—	10	—	KX867192-7201
	*Euphrosinella* cf. *cirratoformis* (MB2)		—	3	—	KX867202-7204
	*Euphrosinopsis* cf. *antarctica* (MB)	no	—	2	—	KX867205-7206
*Glycera capitata* (Glyceridae)	*Glycera* sp. (MB1)	yes	4	15	KX867392-7395	KX867232-7246
	*Glycera* sp. (MB2)		9	17	KX867396-7404	KX867247-7263
Hesionidae sp. A (Hesionidae)	Hesionidae sp. (MB1)	yes	5	24	KX867421-7425	KX906540-6562
	Hesionidae sp. (MB2)		8		KX867426-7433	
*Lumbrineris kerguelensis-cingulata* (Lumbrineridae)	*Lumbrineris kerguelensis-cingulata* (MB1a)	no	—	5	—	KX867315-7319
	*Lumbrineris kerguelensis-cingulata* (MB1b)		—	1	—	KX867320
	*Lumbrineris kerguelensis-cingulata* (MB1c)		—	5	—	KX867321-7325
	*Lumbrineris kerguelensis-cingulata* (MB1d)		—	2	—	KX867326-7327
	*Lumbrineris kerguelensis-cingulata* (MB1e)		—	1	—	KX867328
	*Lumbrineris kerguelensis-cingulata* (MB1f)		—	2	—	KX867329-7330
*Maldane sarsi* (Maldanidae)	*Asychis amphiglyptus* (MB)	no	—	4	—	KX867171-7174
	*Eupraxillella* cf. *antarctica* (MB)	no	—	5	—	KX867207-7211
	*Maldane sarsi antarctica* (MB)	yes	—	2	—	KX867345-7346
	Maldanidae sp. (MB)	no	—	1	—	KX867347
	*Praxillella* sp. (MB)	no	—	1	—	KX867348
*Aglaophamus trissophyllus* (Nephtyidae)	*Aglaophamus* cf. *trissophyllus* (MB1a)	yes	3	8	KX867389-7391	KX867140-7147
	*Aglaophamus trissophyllus* (MB1b)		2		KX867381-7382	
	*Aglaophamus* cf. *trissophyllus* (MB1c)		1		KX867383	
	*Aglaophamus* sp. (MB2)		3	22	KX867384-7386	KX867117-7121
	*Aglaophamus* sp. (MB3)		2		KX867387-7388	KX867123-7139
	*Aglaophamus* sp. (MB4)	no	—	1	—	KX867122
*Aricidea simplex* (Paraonidae)	*Aricidea simplex* (MB)	no	—	9	—	KX867162-7170
	*Aricidea* cf. *belgicae* (MB1)	yes	—	10	—	KX867148-7157
	*Aricidea* cf. *belgicae* (MB2)		—	2	—	KX867158-7159
	*Aricidea* cf. *belgicae* (MB3)		—	1	—	KX867160
	*Aricidea* cf. *pulchra* (MB)	no	—	1	—	KX867161
*Harmothoe fuligineum* (Polynoidae)	*Harmothoe fuligineum* (MB)	no	6	15	KX867415-7420	KX867264-7278
*Macellicephala* sp. A (Polynoidae)	*Macellicephala* sp. (MB1)	yes	3	9	KX867445-7447	KX867369-7377
	*Macellicephala* sp. (MB2)		1	3	KX867448	KX867378-7380
*Macellicephaloides* sp. B (Polynoidae)	*Macellicephaloides* sp. (MB1a)	no	—	12	—	KX867331-7342
	*Macellicephaloides* sp. (MB1b)		—	2	—	KX867343-7344
*Scalibregma inflatum* (Scalibregmatidae)	*Scalibregma* sp. (MB1)	yes	6	14	KX867449-7454	KX867349-7362
	*Scalibregma* sp. (MB2)		1	1	KX867455	KX867363
	*Scalibregma* sp. (MB3)		3	4	KX867455-7458	KX867364-7367
*Laonice weddellia* (Spionidae)	*Laonice weddellia* (MB)	no	7	23	KX867437-7444	KX867292-7314
	*Laonice* cf. *antarctica* (MB)	no	2	6	KX867434-7435	KX867279-7284
	*Laonice* cf. *vieitezi* (MB)	no	0	5	—	KX867285-7289

For *Aricidea belgicae, Euphrosinella cirratoformis* and *Maldane sarsi* no COI sequences were collected and thus the evidence for cryptic species is purely from 16S sequence analysis. The K2P intraspecific and interspecific variation recorded was variable for each morphospecies. For example in *E. cirratoformis*, the average K2P between the two clades was 6.28%, much greater than the 2.31% difference between the three *Aricidea* clades (figure 6). Despite these lower values between the *A. belgicae* clades, these were still considered potential cryptic species as the genetic distance between *A. belgicae* and different morphotypes within the same genus were similar (electronic supplementary material, table S1).

### 3.2. Evidence of cryptic species in the COI phylogeny only

The recognition of potential cryptic species as opposed to intraspecific variation became more complex when single gene COI and 16S analyses produced different results (scenario 2 described above). Such difference between COI and 16S has previously been recorded in the Antarctic polychaete *Austrolaenilla antarctica* [20]. In our study, *Aglaophamus trissophyllus* COI analysis revealed the presence of five different clades, while 16S only revealed two of these (table 2). After examining the inter- and intra-clade K2P distances across the five COI clades, three clades were considered to be a potential species complex (MB1a–c), as defined in our methods, rather than cryptic species. The interspecific differences between this species complex and the two remaining *Aglaophamus* sp. clades (MB2–3) identified from COI analysis ranged from 11 to 14% (electronic supplementary material, table S1), thus these were considered to be cryptic species (table 2). When the interspecific and intraspecific variation of the corresponding 16S was measured there was no clear barcoding gap, although the average interspecific distance was greater than the intraspecific, 2.28% compared with 0.25%, (figure 6).

Similarly for Hesionidae sp. A, differences in the number of clades produced by COI and 16S were not coherent. Once more it was 16S that produced the more conserved diversity results, with only COI providing evidence for cryptic species. The corresponding 16S sequences from the specimens within different COI clades are labelled in figure 4, demonstrating that without COI for comparison these cryptic species would not have been identified. Thus, for those specimens morphologically identified as Hesionidae sp. A with no COI sequence it was not possible to assign them to cryptic clades observed in COI and they are labelled Hesionidae sp. (MB).

### 3.3. Undetermined clades from 16S analysis

In two of the target species, *Lumbrineris kerguelensis-cingulata* and *Chaetozone* sp. A, phylogenetic and distance analyses were unable to resolve whether the clades formed were potential cryptic diversity or a result of the morphospecies being a species complex [47]. This uncertainty was a combination of tree topography, overlapping inter- and intra-clade K2P distances and lack of COI data (electronic supplementary material, table S1), which may have revealed greater genetic distances (figures 5 and 6). Previous investigations have uncovered subtle morphological variations within these genera and have also suggested the existence of species complexes [47–49]. Furthermore, many of the specimens were incomplete. Given the high genetic diversity and associated taxonomic uncertainties, it is perhaps impossible to consider them as cryptic species as the missing material may contain morphological features that deem them separate morphospecies. Thus, a conservative approach to estimating species diversity was taken. Given the high intraspecific clade variation (up to 15.54%) in *Lumbrineris kerguelensis-cingulata*, it is likely that this complex contained a mixture of different morphospecies and possibly cryptic species. However, given the material and data available, we are unable to resolve this in this study.

### 3.4. No evidence of cryptic species

There was no convincing evidence of cryptic diversity in six of the original target species. For these species, the majority of individuals were contained within a single clade with generally low K2P distances in pairwise comparisons. Less than 1% intraspecific variation on average was recorded for both COI and 16S, in five of these six species (figure 6). These included well-known Antarctic species; *Laonice weddellia* and *Harmothoe fuligineum* as well as the two undescribed Acrocirridae species; *Flabelligena* sp. A and *Flabelligena* sp. B. For *Aricidea simplex* and the undescribed Polynoidae species *Macellicephaloides* sp. B only 16S sequences were obtained. Given the faster mutation rate of COI, it is not beyond possibility that if this gene was obtained, greater variation would have been recorded. However, using the data available there is no suggestion of cryptic diversity within these clades.

### 3.5. GenBank comparisons

By comparison to publicly available sequences on GenBank the Antarctic specimens morphologically identified as the described Northern Hemisphere species, *Glycera capitata* and *Scalibregma inflatum,* were shown to be cryptic species. In the case of *Glycera* sp. (MB2), these sequences matched *Glycera ‘*clade II’ sequences on GenBank, a previously identified cryptic species of *G. capitata* found within the Weddell Sea [21]. It also confirmed the presence of *Harmothoe fuligineum* and *Aglaophamus trissophyllus* within the BIOPEARL and JR275 samples by comparison to GenBank sequences obtained from other Antarctic specimens. Several larval DNA sequences collected from specimens in the Ross Sea matched BIOPEARL morphospecies, including two *Laonice* species and Hesionidae sp. A [50,51]. The comparison to sequences on GenBank also revealed genetic differences between Antarctic subspecies and their Northern parent species. In the case of *Maldane sarsi*, following DNA barcoding of the BIOPEARL specimens and taxonomic discussion, these individuals were assigned to the subspecies *M. sarsi antarctica.* This questions the usefulness of its subspecies status, if the subspecies are both genetically distinct from and located a significant distance from its parent species, we query whether they are actually separate morpho- or cryptic species.

### 3.6. Secondary morphological findings

As listed in table 2, secondary morphological analysis led to some individuals being reassigned to different morphospecies owing to initial taxonomic oversight and thus the genetic differences found during phylogenetic analysis were not a result of ‘true’ cryptic diversity but rather primary misidentification. In total, 10 additional morphospecies were identified within five of the original morphospecies. These were found in the following species; *Laonice weddellia,* which contained two other Antarctic *Laonice* species including *Laonice* cf. *vieitezi* and *L.* cf. *antarctica*; *Maldane sarsi* which contained two unidentifiable species as well as *Asychis amphiglyptus* and *Eupraxillella* cf. *antarctica*; *Aricidea simplex* which contained mostly *A. belgicae* specimens and a single *A.* cf. *pulchra* specimen*; Euphrosinella cirratoformis* which contained several *Euphrosinopsis* cf. *antarctica* specimens; and *Aglaophamus trissophyllus* which also contained an unidentifiable *Aglaophamus* species (*Aglaophamus* sp. (MB4)).

## 4. Discussion

Using DNA barcoding, the number of species within our subsample increased by 233% from 15 to 35. This was a result of 10 additional cryptic species identified within half of the target species and 10 additional morphospecies uncovered during secondary morphological examination. This suggests that using routine morphological identification, with the rather inadequate taxonomic sources available, collectors are missing an astonishing amount of Southern Ocean biodiversity. In each case of suspected cryptic species, the results were discussed with specialist taxonomists for specific polychaete families and previous taxonomic findings were considered. The efficiency of detecting cryptic diversity would be much greater if general rules could be applied. For example, a minimum of 10 times the average intraspecific variation between clade differences has been suggested as a rule of thumb for identifying cryptic species [52]. This method was used to identify provisional species in a major polychaete barcoding project [29]. Using the COI marker, Carr *et al*. [29] detected cryptic species in more than 25% of the species investigated with on average 16.5% sequence divergence between species and 0.68% within species. In our study, the interspecific variation between cryptic clades for COI sequences was on average at least 20 times greater than the intraspecific variation (figure 6). For 16S, this difference was lower ranging from 9 to 28 times more interspecific than intraspecific variation. These results indicate that our ability to apply strict rules to the identification of cryptic species within polychaetes is limited. A lack of evidence for a global DNA barcoding gap in Annelida was also recorded in Kvist [53], who evaluated over 70 million pairwise genetic comparisons using the Automated Barcoding Gap Discovery software [54]. Where possible, a number of genes and phylogenetic analyses should be used when determining the presence of cryptic species.

These data contribute to the growing body of evidence which suggests that the Antarctic benthos is far more species rich than previously thought [2,55]. The first major review of Southern Ocean deep-sea diversity by Brandt *et al*. [56] noted that a high proportion of species were considered new to science, many of which were also considered to be Southern Ocean endemics and rare. At the time, these data were sufficient to provide a sound basis to conserve the Southern Ocean as a fragile marine environment. At the same time, there was also a growing number of genetic datasets presenting evidence for cryptic species, in addition to ongoing speciation in some taxa [57]. As previously stated, cryptic diversity has now been documented in all major invertebrate taxa within the Southern Ocean; however, most of these studies only investigate a single genus or species. An exception to this is the study by O'Loughlin *et al*. [58] investigating the genetic diversity of 28 holothurian morphospecies. Within this study, an average of three divergent lineages were uncovered in 17 of the 28 morphospecies targeted, thereby significantly increasing species richness.

The presence of cryptic species among Antarctic fauna suggests that genetic differentiation between populations may have been driven by multiple factors. The aforementioned glacial history of Antarctica creating physical barriers between populations and thus preventing reproductive exchange is currently believed to be the most likely explanation of cryptic speciation [2]. It has often been predicted that cryptic species result from, and are more abundant in, widely distributed species with direct development or short-lived larvae [59]. Given the abundance of invertebrate species lacking a planktonic larval phase in Antarctica [60], it could be suggested that cryptic species may be more abundant in Antarctica as a result of the combined influences of both environmental and biological factors. With the exception of a few chemosynthetic species (e.g. Glover *et al*. [61], McHugh [62], Van Dover *et al*. [63]) our knowledge of the reproductive traits of deep-sea polychaetes is perhaps too limited to consider such traits as potential speciation drivers. For most polychaetes, their reproductive traits (e.g. whether species are brooders or spawners, if they have larval stages and whether these are feeding or non-feeding) are generally classified at the family level from studies based on shallow-water species. In our study, there is a mixture of reproductive modes within species containing cryptic clades [64], and so we are unable to predict whether family-level traits could have promoted genetic divergence. These findings are consistent with that of Nygren [28]; in this review it was concluded that no generalizations can be made about which type or types of polychaetes could be more likely to contain cryptic species given their existence across varying life histories and environments.

The geographical and depth distribution of the different cryptic clades and potential species identified within this study are yet to be investigated (Brasier *et al.* [65]), although some biogeographic implications of these data are already evident. The presence of cryptic Antarctic clades within morphospecies described from the Northern Hemisphere including *Glycera capitata, Scalibregma inflatum* and *Maldane sarsi* indicates that we should be questioning the current ‘usual’ identifications of cosmopolitan polychaete species. To address such questions thoroughly would require the phylogenetic analysis incorporating DNA barcodes from type material, or if unavailable, specimens collected at their type locality, including Greenland (*Glycera capitata*)*,* Norway (*Scalibregma inflatum*) and Sweden *(Maldane sarsi*). Genetic evidence for cosmopolitan polychaetes does exist. For example, the vestimentiferan tube worm *Sclerolinum contortum* has shown genetic consistency in the COI gene among specimens collected from both polar regions and the Gulf of Mexico [66]. However, for the majority of Antarctic species investigated widespread distribution and circumpolarity is rarely recorded and multiple species with more restricted ranges are more common [17,23,67–69].

With the increased abundance of geo-referenced DNA sequences generated from barcoding studies, the assessment of species distributions could not only provide insight into the drivers of this cryptic diversity but also assist in marine management and monitoring in regions undergoing ecosystem change, such as Antarctica. Furthermore, these data provide the baseline for future investigations into the importance of cryptic species at a functional level, species response to environmental variability and its impact on ecosystem function and services. The lack of morphological differences between them could suggest that cryptic clades remain functionally similar. However, in some taxa physiological experiments have revealed functional variability between cryptic species. For example, different growth rates have been recorded in cryptic clades of the phytoplankton *Chaetoceros socialis* under different temperature conditions [70] and in the marine protist *Oxyrrhis marina* when exposed to different salinities [71]. The dominance, coexistence and interspecific competition between cryptic species may also be variable under changing conditions as indicated by experiments on *Rhabditis marina* [72]. Differences in the natural products produced by cryptic clades of the bryozoa *Bugula neritina* have also been recorded [73]. Although such experiments on living specimens may not be possible for deep-sea polychaetes, if species-specific biological trait data were to be collected from preserved material this could provide insight into the importance of diversity at the functional level, i.e. role of diversity and cryptic diversity in maintaining ecosystem services in rapidly changing marine environments.

The comparison of DNA sequences in this project uncovered an underestimation of species diversity as a result of the presence of cryptic species, in addition to errors in morphological identification, which may be an additional contributing factor. Within five of the morphologically identified target taxa multiple morphospecies were identified during secondary morphological analysis. Previous misidentifications of several species within this study could have resulted from multiple factors. For example, the soft bodies of polychaetes can be easily damaged during sample processing. The resulting presence of incomplete specimens, especially those that have lost critical identifying features, reduces the accuracy of species identification [74]. An example of the latter in our study was found in *Aricidea*, where the median antenna present on *Aricidea simplex* (distinguishing it from *A. belgicae*) was detached in some cases leading to false identification prior to DNA sequencing. As seen for *Laonice weddellia*, genetically distinct clades were actually specimens of *Laonice* cf. *vieitezi*, which was described after the specimens in our study were first morphologically identified [74]. Finally, incorrect taxonomic decisions inherently associated with the processing of large numbers (around 20 000) of specimens in a limited time frame (ultimately defined by funding) may have also been an influencing factor. Furthermore, the EBS used to collect specimens targets smaller individuals, thus many morphospecies were juveniles, including *Aglaophamus trissophyllus*, with ontogenetic differences to their adult counterparts collected in the AGT. The secondary morphological examination after sequencing is therefore vitally important to prevent false positive results and an overestimation of ‘true’ cryptic diversity. The morphological differences identified during secondary analysis following DNA barcoding highlights that DNA barcoding should be considered a complementary method of species identification for diversity investigations rather than a replacement [75]. This is most important in species or families lacking reference sequences on public databases as DNA barcodes would not be able to connect individuals to a known species. Increased numbers of unidentified specimens limits the use of their sequences in future studies such as biogeography or for management tools.

The complementary results between the two mitochondrial genes 16S and COI in several morphospecies demonstrates that despite the slower evolutionary rates of 16S it can in many cases fulfil the barcode criteria set out by Hebert *et al*. [31]. Similar results have also been observed in other taxa including amphibians [33], crustaceans [18] and nudibranchs [76]. A greater abundance of publicly available 16S sequences compared to COI for Antarctic invertebrates was noted in Grant & Linse [6]. Furthermore, as recorded within family-level polychaete studies the retrieval of 16S is often more successful as seen for the Hesionidae [77] and Nephtyidae [78]. Thus, 16S should not be initially viewed as an inferior barcoding gene to COI. In many species, 16S provided greater specimen coverage without underestimating species diversity. However, the collection of both genes, at least from clade representatives, can aid in the discrimination between high intraspecific variation and potential cryptic species as observed for Hesionidae sp. A and *Aglaophamus trissophyllus*.

In conclusion, we can confidently accept the overarching hypothesis of this study as our data reveal that current levels of Antarctic polychaete diversity are vastly underestimated. There are nearly 800 species records of polychaetes within the Register of Antarctic Marine Species (RAMS), which have been documented in Antarctic waters [79]. The combined factors of undersampling, undescribed species and cryptic species suggest that true Antarctic species diversity for polychaetes will be far in excess of this figure. As for the general prevalence of cryptic species, given the uncertainties associated with the detection of cryptic species, including scientific opinion regarding their definition and identification, intraspecific variability and phylogenetic understanding, it is perhaps impossible to suggest the total prevalence of cryptic species within the currently recorded Antarctic polychaetes. In agreement with previous studies [28,29], there appear to be no patterns in cryptic diversity across families. However, other factors remain to be investigated, including biogeography and functional traits, which should be a primary focus of future barcoding projects. The results of this study contribute to the ongoing research effort to document, describe and understand the diversity, biogeography and functionality of Antarctic marine fauna. Such data are of the utmost importance for effective research-driven ecosystem-based management of the rapidly changing Antarctic marine ecosystem.

## Supplementary Material

Table S1 Minimum and maximum K2P pairwise comparison values (%) by family. Including intraspecific (MB, MB#) and intraclade (MB#a) comparisons as well as interspecific and interclade comparisons

## Data accessibility

All new sequence data have been deposited in the GenBank Database, accession numbers by species are listed in table 2.
