# Correction to ‘DNA barcoding uncovers cryptic diversity in 50% of deep-sea Antarctic polychaetes’

**DOI:** 10.1098/rsos.160937

**Published:** 2017-01-04

**Authors:** Madeleine J. Brasier, Helena Wiklund, Lenka Neal, Rachel Jeffreys, Katrin Linse, Henry Ruhl, Adrian G. Glover

The submitted correction is an updated version of figure 1. In the correct figure, shown below, the following has been changed from the figure in the publication:

**Figure 1.**
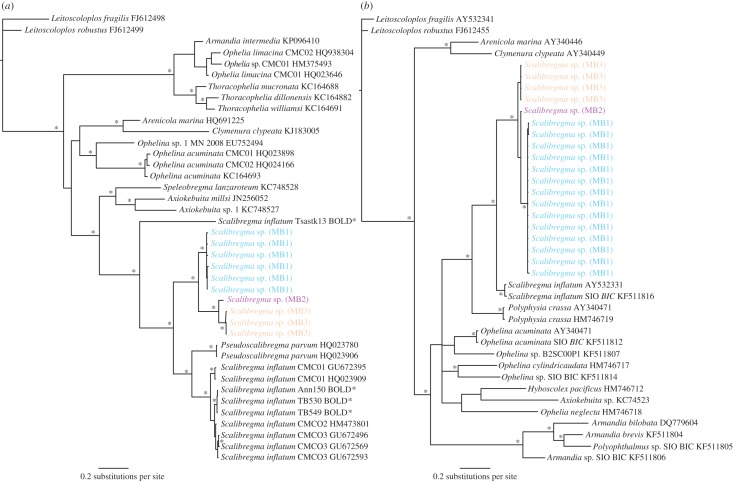
Phylogenetic tree of Scalibregmatidae from Bayesian analysis using COI (*a*) and 16S (*b*). An example of results ‘scenario 1’, evidence for cryptic diversity in COI and 16S genes, cryptic species *Scalibregma* sp. (MB1), (MB2) and (MB3). Outgroup: *Leitoscoloplus fragilis* and *L. robustus* (Orbiniidae), asterisk indicates significant node values (more than 95%) for Bayesian posterior probabilities. BOLD* indicates sequences obtained from a private BOLD database.

In the COI tree (figure 1*a*), the sequence in purple has been renamed *Scalibregma* sp. (MB2) (from (MB1) in blue).

In the 16S tree (figure 1*b*), the sequences labelled *Scalibregma* sp. (MB3) have been recoloured to orange (from black).

