# Supplementary material for: DNA barcoding uncovers cryptic diversity in 50% of deep-sea Antarctic polychaetes
Source: R Soc Open Sci. 2016 Nov 2;3(11):160432. doi: 10.1098/rsos.160432 (PMC5180122; doi:10.1098/rsos.160432)
Supplement: Table S1 Minimum and maximum K2P pairwise comparison values (%) by family. Including intraspecific (MB, MB#) and intraclade (MB#a) comparisons as well as interspecific and interclade comparisons [file rsos160432supp1.pdf]

Family	Morphospecies (MB), Cryptic species (MB#) or Clade (MB#a)	COI						16S						
		**Intraspecific/ Intraclade K2P %**	**Interspecific/ Interclade K2P %**					**Intraspecific/ Intraclade K2P %**	**Interspecific/ Interclade K2P %**					
Acrocirridae	1. *Flabelligena* sp. A (MB)	0.00-1.90	1.	2.				0.75*	1.	2.				
	2. *Flabelligena* sp. B (MB)	0.00-1.88	24.83-31.06					0.00-1.33	24.68-26.46					
Cirratulidae	*1. Chaetozone* sp. (MB1a)							0.00-0.019	1.	2.	3.			
	2. Chaetozone sp. (MB1b)							**	2.72-4.35					
	3. Chaetozone sp. (MB1c)							0.27*	5.70-7.22	4.53-4.88				
Euphrosinidae	1. *Euphrosinella* cf *cirratoformis* (MB1)							0.00-2.42	1.	2.	3.			
	2. *Euphrosinella* cf *cirratoformis* (MB2)							0.27-0.28	5.50-7.53					
	3. *Euphrosinopsis* cf *antarctica* (MB)							0.62*	21.11-27.44	20.41-24.83				
Glyceridae	1. *Glycera* sp. (MB1)	0.00-0.44	1.	2.				0.00-2.74	1.	2.				
	2. *Glycera* sp. (MB2)	0.00-0.30	17.12-18.05					0.00-1.60	8.17-12.80					
Hesionidae	1. Hesionidae sp. (MB1)	0.15-0.51	1.	2.				0.00-2.97						
	2. Hesionidae sp. (MB2)	0.00-1.01	10.59-12.41											
Lumbrineridae	*1. Lumbrineris kerguelensis-cingulata* (MB1a)							0.00-1.42	1.	2.	3.	4.	5.	6.
	2. Lumbrineris kerguelensis-cingulata (MB1b)							**	9.45-11.61					
	3. Lumbrineris kerguelensis-cingulata (MB1c)							0.00-0.36	9.22-11.74	5.62-7.63				
	4. Lumbrineris kerguelensis-cingulata (MB1d)							1.46*	9.35-11.97	6.24-6.70	4.38-5.88			
	5. Lumbrineris kerguelensis-cingulata (MB1e)							**	10.40-12.50	6.11*	4.57-6.10	4.86-4.88		
	6. Lumbrineris kerguelensis-cingulata (MB1f)							0.00-0.36	10.35-15.54	5.77-7.56	3.91-5.54	4.21-5.22	3.91-5.21	
Maldanidae	1. *Asychis amphiglyptus* (MB)							0.27-2.05	*1.*	*2.*	*3.*	*4.*	*5.*	
	2. *Eupraxillella* cf *antarctica* (MB)							0.28-4.11	35.11-49.94					
	3. *Maldane sarsi antarctica* (MB)							0.26*	15.31-16.82	30.51-36.26				
	4. Maldanidae sp. (MB)							**	35.62-40.50	20.24-24.39	37.72-33.27			
	5. *Praxillella* sp. (MB)							**	39.39-40.48	23.08-24.83	37.47-37.65	10.81*		
Nephtyidae	1. *Aglaophamus* cf *trissophyllus* (MB1a)	0.00-0.18	1.	2.	3.	4.	5.	0.00-1.67	*1. 2. 3.*	*4. 5.*	*6.*			
	2. *Aglaophamus trissophyllus* (MB1b)	0.92*	5.33-5.75											
	3. *Aglaophamus* cf *trissophyllus* (MB1c)	**	4.54-4.74	5.54-6.13										
	4. *Aglaophamus* sp. (MB2)	0.16-0.18	13.55-14.01	13.09-13.34	13.31-13.54			0.00-0.80	1.63-3.38					
	5. *Aglaophamus* sp. (MB3)	2.12*	11.56-13.06	11.52-12.97	12.45-13.08	9.64-13.32								
	6. *Aglaophamus* sp. (MB4)							**	5.58-6.90	5.53-6.13				

Family	Species	COI intra	COI 1	COI 2	COI 3	16S intra	16S 1	16S 2	16S 3	16S 4	16S 5
Paraonidae	1. *Aricidea simplex* (MB)					0.00-1.45	*1.*	*2.*	*3.*	*4.*	*5.*
	2. *Aricidea* cf *belgicae* (MB1)					0.00-0.61	18.70-22.63				
	3. *Aricidea* cf *belgicae* (MB2)					0.27	18.34-21.63	0.82-1.54%			
	4. *Aricidea* cf *belgicae* (MB3)					**	19.16-21.22	2.46-3.07%	2.45-2.78		
	5. *Aricidea* cf *pulchra* (MB)					**	19.43-22.37	4.18-5.05%	3.89-4.18	3.05*	
Polynoidae	1. *Harmothoe fuligineum* (MB)	0.00-1.47	1.	2.	3.	0.00-2.28	1.	2.	3.	4.	5.
	2. *Macellicephala* sp. (MB1)	0.15-0.73	27.03-30.86			0.00-1.08	34.52-47.89				
	3. *Macellicephala* sp. (MB2)	**	25.47-26.85	12.58-12.84		0.00-0.56	31.58-36.45	2.74-4.30			
	4. Macellicephaloides sp. (MB1a)					0.00-4.10	34.61-41.81	36.46-54.35	35.29-43.05		
	5. Macellicephaloides sp. (MB1b)					0.58*	36.90-41.08	38.05-45.40	37.07-38.17	0.87-2.36	
Scalibregmatidae	1. *Scalibregma* sp. (MB1)	0.14-1.48	1.	2.	3.	0.00-2.86	1.	2.	3.		
	2. *Scalibregma* sp. (MB2)	**	13.99-14.53			**	4.92-6.66				
	3. *Scalibregma* sp. (MB3)	0.00-1.46	14.35-15.33	6.51-6.77		0.00-1.56	6.66-9.82	2.13-2.44			
Spionidae	1. *Laonice weddellia* (MB)	0.00-1.32	1.	2.		0.00-1.44	1.	2.	3.		
	2. *Laonice* cf *antarctica* (MB)	0.17*	22.54-24.10			0.00-0.27	16.74-19.07				
	3. *Laonice* cf *vieitezi* (MB)					0.00-1.70	14.84-17.28	16.10-18.44			

Table S1 Minimum and maximum K2P pairwise comparison values (%) by family. Including intraspecific (MB, MB#) and intraclade (MB#a) comparisons as well as interspecific and interclade comparisons indicated by colour. Where blue = morphospecies comparisons, purple = cryptic species comparisons and green = within species clade comparisons for both the COI (left) and 16S (right) markers. * indicates no range available because only one pairwise comparison, ** indicates no intra pairwise comparison as only one sequence available for the relative species/clade. Note that for some families/species no COI data was obtained and in the case of Hesionidae sp. A and *Aglaophamus* spp. a greater number of species/clades were obtained from COI data than 16S.
